# Bacterial coinfections with coronavirus disease 2019 (COVID-19)

**DOI:** 10.1017/ash.2021.187

**Published:** 2021-11-10

**Authors:** Glen Huang, Daisuke Furukawa, Bryant D. Yang, Brian J. Kim, Arthur C. Jeng

**Affiliations:** 1 Division of Infectious Diseases, Department of Medicine, University of California – Los Angeles, Los Angeles, California; 2 Department of Pharmacy, Olive View - University of California Los Angeles Medical Center, Sylmar, California; 3 Division of Infectious Diseases, Department of Medicine, Olive View - University of California Los Angeles Medical Center, Sylmar, California

## Abstract

**Background:**

The pandemic caused by severe acute respiratory coronavirus virus 2 (SARS-CoV-2) has dramatically increased cheshospitalizations, and it is often difficult to determine whether there is a bacterial or fungal coinfection at time of presentation. In this study, we sought to determine the rates of coinfection and utilization of antibiotics in SARS-CoV-2 disease.

**Methods:**

Retrospective chart review of patients hospitalized with COVID-19 pneumonia from April 13, 2020, to July 14, 2020.

**Results:**

In total, 277 patients were hospitalized for COVID-19 pneumonia during this period. Patients that received antibiotics within 48 hours of presentation were more likely to be febrile (59.3% vs 41.2%; *P* = .01) and to have leukocytosis (23.9% vs 5.9%; *P* < .01) and were less likely to have a procalcitonin level <0.25 ng/mL (58.8% vs 74.5%; *P* = .04). In total, 45 patients had positive blood cultures collected during hospitalization, 16 of which were clinically significant. Of the clinically significant blood cultures, 5 were collected <48 hours of admission. Moreover, 18 sputum cultures were clinically significant, 2 of which were collected within 48 hours of admission.

**Conclusion:**

Bacterial and fungal coinfections in COVID-19 appear to be rare on presentation; thus, this factor may be a good target for enhanced antibiotic stewardship.

Since the start of the pandemic, coronavirus disease 2019 (COVID-19) has infected millions of patients and has caused significant mortality worldwide.^1^ Many of these patients present with signs and symptoms that are the same as that of bacterial pneumonia, prompting empiric antibiotic usage. The current Infectious Diseases Society of America/American Thoracic Society guidelines recommend empiric antimicrobial therapy in patients with influenza infection and radiographic evidence of community-acquired pneumonia in both the inpatient and outpatient setting.^2^ However, data on the clinical circumstance on antibiotic utilization in COVID-19 remain sparse. A review of the literature found that 5%–27% of COVID-19 cases had an associated secondary infection,^2^ and a Virginia study noted a significant increase in ceftriaxone and azithromycin use in the medical intensive care unit (ICU) during the pandemic.^3^ In this study, we sought to capture the antibiotic utilization and rate of secondary infection in patients with COVID-19.

## Methods

This study was a retrospective chart review of all adult patients with positive nasopharyngeal SARS-CoV-2 polymerase chain reaction (PCR) assay admitted to Olive View–University of California–Los Angeles Medical Center, a hospital funded by Los Angeles County in California. Only patients suspected of having COVID-19 pneumonia as documented in the medical records were included, and asymptomatic patients who were admitted for an unrelated cause who screened positive with the SARS-CoV-2 PCR were excluded. Information regarding patient demographics, clinical findings at presentation and during hospitalization, laboratory results, and microbiology were collected and managed in Microsoft Excel software (Microsoft, Redmond, WA). Comorbidities were defined based on clinical documentation and *International Classification of Diseases, Tenth Revision* (ICD-10) codes. Laboratory values were recorded as the first values available on presentation. Pneumonia was defined by chart review based on clinical characteristics of patients at presentation. Only commonly pathogenic organisms were considered clinically significant, and organisms that are typically considered as a contaminant or a colonizer (eg, coagulase-negative *Staphylococcus* on blood culture or *Candida* spp on respiratory culture) were not considered clinically significant. Pathogenic coagulase-negative *Staphylococcus* in the blood was defined as patients having received targeted therapy or deemed to be clinically significant based on documentation. These clinical parameters were compared between patients who received empiric antibiotics within 48 hours of admission and those who did not.

Antibiotic utilization was further characterized in detail. Antibiotic delivery was stratified as having started either early (within 48 hours of admission) or late (after 48 hours). The class, specific antibiotics, and days of therapy were abstracted by manual chart review.

### Statistical analysis

Categorical variables were analyzed using the χ^2^ or Fisher exact test. Continuous variables were evaluated using the Student *t* test or the Mann-Whitney *U* test where appropriate.

**Fig. 1.**
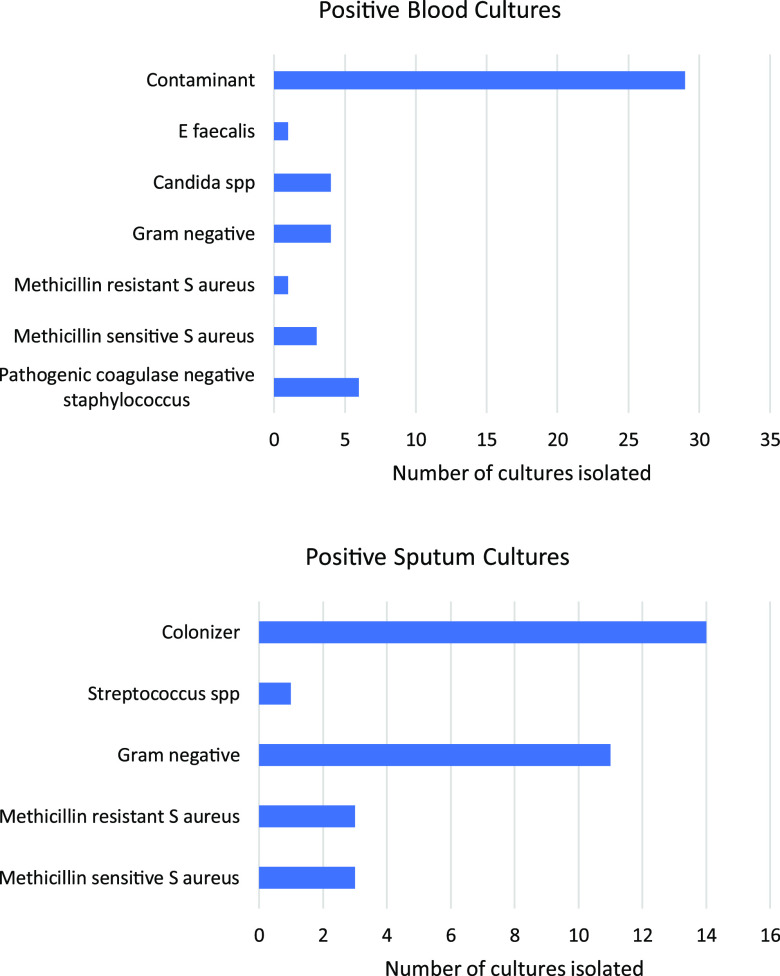
Frequencies of positive blood cultures (top) and positive sputum cultures (bottom).

## Results

Overall, 684 patients with a positive SARS-CoV-2 nasopharyngeal PCR between April 13, 2020, to July 14, 2020, were included. Of these, 277 had been admitted for COVID-19 pneumonia and either died during their hospitalization or were discharged. Also, 51 patients did not receive antibiotics within 48 hours of hospitalization and 226 patients did (Table 1). The overall mean length of stay was 8.5 days (range, 0–75). Individuals who did not receive antibiotics within 48 hours were less likely to be febrile (41.2% vs 59.3%; *P* = .01) and were less likely to have leukocytosis (5.9% vs 23.9%; *P* < .01), neutrophilia (9.8% vs 31.4%; *P* < .01), or lymphopenia (56.9% vs 71.2%; *P* < .01). They were more likely to have chest radiograph findings that were negative for any acute processes (23.5% vs 8.8%; *P* < .01). In terms of inflammatory markers, individuals who were not administered antibiotics within 48 hours of hospitalization had lower mean levels of D-dimer (0.73 vs 0.97; *P* = .04), ferritin (369 vs 801; *P* < .01), and C-reactive protein (73.3 vs 137.7; *P* < .01); they were also more likely to have a procalcitonin level <0.25 ng/mL (74.5% vs 58.8%; *P* = .04). We detected no statistically significant differences in symptoms between the 2 groups. Patients who were not administered antibiotics were less likely to require supplemental oxygen (56.9% vs 88.9%; *P* < .01) or intubation (0% vs 19.9%; *P* < .01) during the admission. They also had a lower in-hospital mortality rate (5.9% vs 18.6%; *P* = .03).

**Table 1.** Characteristics of Patients With COVID-19

Characteristic	Did Not Receive Antibiotics Within 48 Hours of Hospitalization (n = 51), No. (%)^a^	Received Antibiotics Within 48 Hours of Hospitalization (n = 226), No. (%)^a^	*P* Value
**Demographics**			
Age, y	54.5 (46–63.25)	56 (46–69)	.21
No comorbidities	10 (19.6)	51 (22.6)	.65
Hypertension	23 (45.1)	94 (41.6)	.65
Diabetes mellitus	22 (43.1)	78 (34.5)	.25
Coronary artery disease	3 (5.9)	11 (4.9)	.73
Congestive heart failure	6 (11.8)	6 (2.7)	.01
Chronic kidney disease	6 (11.8)	29 (12.8)	.84
End-stage kidney disease	2 (3.9)	10 (4.4)	.87
Lung disease	4 (7.8)	16 (7.1)	.77
Cirrhosis	2 (3.9)	5 (2.2)	.62
From skilled nursing facility or long-term acute-care facility	6 (11.8)	42 (18.6)	.25
**Clinical presentation**			
Patient febrile within first 24 h	21 (41.2)	134 (59.3)	.01
White blood cells ×10^9^ cells/L	6.4 (5.3–7.6)	10.2 (6.2–10.7)	<.01
Presence of leukocytosis (white blood cells >10.5 × 10^9^ cells/L)	3 (5.9)	54 (23.9)	<.01
Presence of neutrophilia (neutrophils >8 × 10^9^ cells/L	5 (9.8)	71 (31.4)	<.01
Presence of lymphopenia (lymphocytes <0.8 × 10^9^ cells/L)	29 (56.9)	161 (71.2)	.04
D-Dimer (mcg/mL)^b^	0.73 (0.48–1.41)	0.97 (0.58–1.85)	.04
Lactate dehydrogenase (U/L) ^a,b^	277.5 (193–361)	329 (243.3–329)	.09
Ferritin (mcg/L)^b^	369 (209.5–607.3)	801 (415.5–1499.3)	<.01
C-reactive protein (mg/L)^b^	73.3 (30.3–163.7)	137.7 (69.2–200.8)	<.01
Procalcitonin (ng/mL)^b^	0.09 (0.05–0.11)	0.15 (0.08–0.41)	<.01
Procalcitonin <0.25 ng/mL^b^	38 (74.5)	133 (58.8)	.04
Procalcitonin 0.26-0.5 ng/mL ^b^	1 (2)	33 (14.6)	<.01
Procalcitonin > 0.5 ng/mL^b^	2 (3.9)	47 (20.8)	<.01
Lobar findings on chest radiograph	5 (9.8)	30 (13.3)	.64
Chest radiograph negative for acute process	12 (23.5)	20 (8.8)	<.01
Outcomes			
Supplemental oxygen required during hospitalization	29 (56.9)	201 (88.9)	<.01
Intubation required during hospitalization	0	45 (19.9)	<.01
Duration of hospitalization, d	2 (1–4)	6 (3–12.25)	<.01
In-hospital mortality	3 (5.9)	42 (18.6)	.03

a Continuous variables are expressed as median (interquartile range).

b First value obtained on admission.

In total, 45 (16.2%) patients had positive blood cultures during their hospitalization (Fig. 1). Among these patients, 16 (36.6%) cultures were clinically significant, and 5 were collected <48 hours after admission. The median time to collection of blood culture for gram-negative bacteremia was 9.5 days (range, 0–55), and the median time to collection of blood culture for *Candida* spp fungemia was 6 days (range, 14–24). For patients with positive sputum cultures (Fig. 1), 18 (56.3%) cultures were clinically significant, of which 2 were collected <48 hours after admission. The median time to collection of positive gram-negative sputum culture was 17 days (range, 1–30).

Of the antibiotics initiated within 48 hours, azithromycin was the most used agent, followed by ceftriaxone. Azithromycin monotherapy was used in 43 patients (19%). Anti-pseudomonal and anti–methicillin-resistant *Staphylococcus aureus (*MRSA) antibiotics were prescribed in 13.8% and 11.6% of patients who had antibiotics initiated within 48 hours of admission, respectively, with median durations of 2 days and 1 day, respectively. When an antibiotic was initiated after 48 hours, the most commonly used was vancomycin, followed by cefepime, with median durations of 3 and 5.5 days, respectively (Table 2). In total, the median duration of all antibiotics was 5 days (interquartile range [IQR], 5–6).

**Table 2.** Utilization of Antibiotics Within and After 48 Hours of Hospitalization

Category of Antibiotic	Antibiotics Initiated Within 48 Hours of Hospitalization (n = 226), No. (%)^a^	Median Days on Antibiotics Initiated Within 48 Hours of Hospitalization (IQR), No. (%)^a^	Antibiotics Initiated After 48 Hours of Hospitalization (n = 55), No. (%)^a^	Median Days on Antibiotics Initiated After 48 Hours of Hospitalization (IQR)
Anti-pseudomonal agent	31 (13.7)	2 (1–3)	36 (65.5)	6 (3–10)
Cefepime	14 (6.2)	3 (1.75–6.25)	24 (43.6)	5.5 (3–8.5)
Piperacillin–tazobactam	13 (5.8)	1 (1–2.5)	9 (16.4)	5 (3–8)
Meropenem	5 (2.2)	1 (1–1.5)	6 (10.9)	3 (2.5–4.25)
Aminoglycoside	2 (0.9)	1.5	2 (3.6)	1
Anti-MRSA agent	26 (11.5)	1 (1–2.25)	32 (58.2)	3 (2–6)
Vancomycin	26 (11.5)	1 (1–2.25)	32 (58.2)	3 (2–5)
Linezolid	0		1 (1.8)	8
Daptomycin	0		1 (1.8)	2
Ceftaroline	0		1 (1.8)	1
Narrow-spectrum β-lactam^b^	155 (68.6)	4 (1–5)	17 (30.9)	5 (2–9.25)
Ceftriaxone	151 (66.8)	4 (1–5)	9 (16.4)	4 (1.5–6)
Amoxicillin–clavulanic acid	1 (0.4)		4 (7.3)	3
Amoxicillin	1 (0.4)	5	2 (3.6)	5
Ampicillin			1 (1.8)	2
Ampicillin–sulbactam	1 (0.4)	2	1 (1.8)	4
Cefazolin	1 (0.4)	1	2 (3.6)	8
Cephalexin	2 (0.9)	4	0	
Azithromycin	211 (93.4)	5 (5–5)	1 (1.8)	5
Levofloxacin^c^	2 (0.9)	3	2 (3.6)	2.5
Ciprofloxacin^c^	0		4 (7.3)	4.5 (4–5)

a Continuous variables are expressed as median (interquartile range).

b Narrow-spectrum β-lactams defined as a noncarbapenem β-lactam agent that does not cover pseudomonas or MRSA.

c Fluoroquinolones not included in anti-pseudomonal category.

## Discussion

The current pandemic is unprecedented, with high mortality. Although prescriber utilization of broad-spectrum antibiotics has been documented in the literature,^4^ the circumstances in which they are used have not been well studied. A published study in Michigan analyzed 1,705 patients with COVID-19 and aimed to describe the number of bacterial coinfections. In that study, 56.6% of patients were prescribed early antibiotic therapy, and only 3.5% of patients had confirmed community-onset bacterial infections.^8^ Our study showed similar rates of antibiotic use despite low rates of culture confirmed bacterial coinfections. It appears that patients with clinically significant cultures (either sputum or blood) tended to have cultures collected several days into their hospitalization in our study, suggesting nosocomial acquisition.

Previous studies have noted an increase in antibiotic utilization in both “narrow-” and “broad-spectrum” categories^3,7^; however, these studies do not have patient-level data on the duration and timing with which each antibiotic was prescribed. Not surprisingly, in our study therapeutics targeting community-acquired pathogens (eg, ceftriaxone and azithromycin) were the antibiotics most commonly prescribed within 48 hours of admission. Broad-spectrum agents were still used during this period; however, they were generally de-escalated quickly with a median duration of ˜1 day. At our institution, broad-spectrum agents have prespecified indications. All antibiotic use, however, is reviewed by our antibiotic stewardship team, who then help in the de-escalation of therapy. Our data also suggest that most broad-spectrum antibiotic use occurred later in the hospitalization rather than as empiric therapy on admission.

One explanation of the use of empiric antibiotics in patients is the difficulty in distinguishing between a bacterial process and COVID-19. Recent guidelines have suggested empiric antibiotics for patients with suspected but unconfirmed COVID-19,^5^ and the World Health Organization has recommended empiric antibiotic therapy for severe COVID-19.^6^ Our results indicate that patients were more likely to get antibiotics in the presence of certain clinical parameters such as fevers or leukocytosis; however, these signs are nonspecific and are not specifically characteristic of a bacterial infection.

Our study had several limitations. It was a single-center retrospective study, so our findings may not be generalizable to all hospitals. Not all patients had blood and/or sputum cultures drawn during the hospitalization, making it hard to distinguish the true secondary infection rate. Also, a pneumonia multiplex PCR film array panel was not routinely used for community-acquired pneumonia at our hospital, so we may have underestimated the true incidence of bacterial coinfection. This diagnostic test would have a higher sensitivity in identifying bacteria than routine cultures would. Lastly, rates of antibiotic use may have been overestimated because azithromycin, which was used as a COVID-19–specific therapy early in the pandemic, was included in our analysis as an antibiotic therapy.

Antimicrobial resistance continues to be a global crisis.^9^ COVID-19 presenting similarly to a bacterial process has certainly not aided in efforts to curb antibiotic use. In our study, the rate of concurrent bacterial processes in COVID-19, as defined by culture-positivity, was very low but antibiotic use was widespread. The pandemic therefore raises a unique stewardship opportunity, and future studies are needed to better characterize the utility of antibiotics in the management of patients with COVID-19, so that its unnecessary antibiotic use can be minimized.
